# Extracting Traffic Signage by Combining Point Clouds and Images

**DOI:** 10.3390/s23042262

**Published:** 2023-02-17

**Authors:** Furao Zhang, Jianan Zhang, Zhihong Xu, Jie Tang, Peiyu Jiang, Ruofei Zhong

**Affiliations:** 1 Key Laboratory of 3D Information Acquisition and Application, MOE, Capital Normal University, Beijing 100048, China; 2 Base of the State Key Laboratory of Urban Environmental Process and Digital Modeling, Capital Normal University, Beijing 100048, China; 3 College of Resource Environment and Tourism, Capital Normal University, Beijing 100048, China; 4 Department of Statistics, Uppsala University, 75120 Uppsala, Sweden

**Keywords:** lidar point cloud, object detection, panoramic image, convolutional neural network, projection transformation

## Abstract

Recognizing traffic signs is key to achieving safe automatic driving. With the decreasing cost of LiDAR, the accurate extraction of traffic signs using point cloud data has received wide attention. In this study, we propose combining point cloud and image traffic sign extraction: firstly, we use the improved YoloV3 model to detect traffic signs in panoramic images. The specific improvement is that the convolution block attention module is added to the algorithm framework, the traditional K-means clustering algorithm is improved, and Focal Loss is introduced as the loss function. It shows higher accuracy on the TT100K dataset, with a 1.4% improvement in accuracy compared to the previous YoloV3. Then, the point cloud of the area where the traffic sign is located is extracted by combining the image detection results. On this basis, the outline of the traffic sign is accurately extracted using the reflection intensity, spatial geometry and other information. Compared with the traditional method, the proposed method can effectively reduce the missed detection rate, narrow the range of point cloud, and improve the detection accuracy by 10.2%.

## 1. Introduction

Traffic sign detection is an important part of driverless and assisted driving. Vehicles need to detect traffic signs on the road ahead in order to obtain road information. Automatic detection and accurate identification of road signs from complex scenes is an important guarantee for driving safety, which is of great significance in the field of intelligent driving. Conventional road sign detection involves finding traffic signs in road images obtained from image acquisition systems, which is a typical image recognition problem, usually based on the color [1,2,3], shape [4,5,6], and multi-feature fusion method. The detected area also needs to be recognized by certain algorithms. At present, the mainstream methods of traffic sign recognition include template matching and machine learning, among others.

Image template matching refers to the process of matching the corresponding relationship between the image to be identified and the template image by translation search. Image template calculating the similarity between the image to be detected and the template, which is widely used in the field of computer vision. Wang Y [7] incorporates OCR techniques to template matching, but the recognition effect is greatly affected by occlusion and angle. Qian R [8] proposed a representation based on new geometric shape features, namely the real-time traffic sign detection system based on template matching of multilevel chain code histograms, which have high robustness under different rotations, scales and illuminations.

Machine learning involves extracting target features from the images for training. The classifier derived from machine learning is then used to match the image to be recognized and the recognition results are obtained. Its common features include edge features and their variants HOG [9], Harr [10], and DTB [11], and FFT shape features [12]. The common machine learning classifiers are: Support Vector Machine (SVM), Extreme Learning Machine (ELM), Random Forest (RF), etc. Stallkamp [13] achieved a recognition accuracy of 95.68% in German public data sets, using directional gradient histogram features combined with a linear discriminant analyzer. Boi F [14] developed a shape-based classification model using SVM. Based on SVM, it used grid search and simulated annealing search, making it possible to classify speed limit signs with an accuracy greater than 99%. Most of the early target detection algorithms were constructed based on manual features, which failed to meet the requirements in terms of speed and accuracy despite the use of a large number of complex feature representations and network gas pedals.

With the development of computer hardware, the method of deep learning has been widely used in target detection. Deep learning target detection models can be divided into two categories: one-stage models and two-stage models. The one-stage model converts the border localization problem into a regression problem for one-step training, generating the category and location information through the backbone network. The final detection result can be directly obtained by a single detection, so it has a faster detection speed, such as Overfeat, Yolo [15], and SSD [16]. The training process of the two-stage series algorithms is divided into two parts: generating region proposals and training RPN networks. Compared with the one-stage model, the accuracy is higher, but speed and real-time performance are poor. The common methods include R-CNN [17], Fast R-CNN, Faster R-CNN [18].

The most mainstream method for target recognition is Convolutional Neural Networks (CNN). There are many precedents for using this method for detection. Y. Ma adjusted the convolutional kernel to 3 × 3 size and added dropout and convolutional layers to the AlexNet model, which achieved an accuracy of 96.875% on the German GTSRB public dataset. Ciresan [19] used a multi-column deep convolutional neural network, reaching an accuracy of 99.46% after testing on the GTSRB dataset. Dan C [20] added and subtracted two standard deviation average pixel intensities on RGB three channels and performed histogram equalization to greatly expand the dataset. Then, he combined HOG features and SVM extraction to achieve an accuracy of 99.15% in GTSRB. Yawar Rehman [21] learned features by gathering semantic information, which reduced the use of sliding window in traditional target detection and relied on color changes to find areas with a higher possibility of traffic signs, achieving an accuracy of 100% on the GTSRB dataset. The deep learning method requires prior training of features from a large amount of sample data. Compared with the traditional manual method, accuracy and robustness are greatly improved, making it is less likely to be undetectable due to lighting, angle, and occlusion.

However, these methods rely on visual images that lack 3D depth information and are sensitive to illumination and weather conditions, thus limiting the accuracy of target detection and localization. In the actual scenario of autonomous driving, we are more frequently faced with 3D targets with depth information; using vision only for 2D detection can neither utilize richer spatial information nor meet the engineering needs in complex scenarios. With the development and application of 3D laser scanning technology, point cloud data acquisition has become fast and inexpensive. In addition to the spatial location information and complete geometric structure of the measured target, LiDAR can also provide the reflection intensity, color, texture, and other information of the target. Such comprehensive and diverse feature descriptions provide more possibilities for target ground feature identification. Vision-LiDAR SLAM obtains more information in the face of intense movement, lack of light, or lack of visual features [22]. In HD maps, real-time positioning and obstacle detection cannot be achieved without the support of LiDAR. Lidar technology is expanding into many different applications, especially obstacle [23] detection and object recognition during autonomous driving [24].

LIDAR-based traffic sign extraction is mainly based on spatial geometric features and reflection intensity features. Lipu Zhou [25] proposed a new traffic sign detection and recognition algorithm based on data fusion of camera and lidar, which fused the data obtained by two sensors to improve the robustness of the algorithm. Yokoyama [26] proposed a rod extraction algorithm based on Laplacian smoothing and Principal Components Analysis (PCA), in which the Laplacian operator is used to smooth the data to eliminate noise points and distribution bias of data points in the data, while the PCA method is used to perform feature analysis on the smoothed data segments and detect rod structure targets from them. Since traffic signs are generally made of highly reflective materials with clear visibility, the reflective intensity information in the point cloud data can effectively distinguish the signage from other targets on the road, thus being widely used in traffic sign detection [27,28]. In addition, template-driven methods are also used to detect traffic signage [29,30]; the detection is realized by using the characteristics of high reflection intensity and symmetry of traffic signs.

With the rapid development of artificial intelligence, researchers at home and abroad have tried to apply deep learning from 2D data to 3D data. Qi [31] proposed a network called PointNet, which directly processes point clouds, exploiting their order invariance and rotation invariance, but without the process of extracting local features. However, the generalization ability of the model is limited in complex scenes. Li [32] put forward a simple and general learning framework of point cloud features, PointCNN, which emulates convolutional neural networks to utilize local correlations on point cloud space; the accuracy rate on the ModelNet is 91.7%.

Although LIDAR has many advantages, it is still difficult to extract traffic signs with high precision. There are three reasons for this. The first is that traffic signs are often obscured by other targets. Secondly, signs that have been outdoors for a long time will inevitably become damaged and aged, dulling their reflection characteristics. Finally, in the process of data acquisition, the density of the target, which is far away from the scanning equipment, is low. So far, the accurate extraction of traffic signs from large-scale point cloud data in different scenes has not been realized. In addition, unlike traditional point cloud classification, vehicles will pass through various complicated environments during driving, and it is difficult to accurately identify the targets along the way through traditional means, which suggests that the detection of traffic signs requires improvement.

Many methods have been proposed for detecting and extracting traffic signs from images or LiDAR data. Due to the emergence of deep learning methods, the accuracy and robustness of traffic sign detection in images have reached a very high level. However, in comparison, the classification and extraction algorithms of point clouds are mostly at the theoretical level and the accuracy is relatively low. One of the most important factors is that point cloud data has more features and complex scenes, so it is not easy to extract the correct category from a large number of data points. Up until now, there has been no research that can combine point cloud and image data sources to extract traffic signs. Qi C R [33] used RGB-D data for 3D object detection, using a mature 2D object detector and advanced 3D deep learning for object localization, identifying conical regions in the point cloud combining a deep learning model for detection. However, there is no targeted training for road signs, and the point cloud deep learning method not only needs great computational power but also needs to improve in accuracy and robustness.

To sum up, extracting the range of traffic signs from massive point cloud data is not only extremely time-consuming, but also has a high false detection rate. Furthermore, it depends on manual discrimination in most cases. To address the above issues, this paper proposes a set of methodological processes for traffic sign extraction by combining camera and LIDAR using sensor fusion technology to obtain detection targets using mature and efficient image detection means, then combining them with laser point cloud for fine extraction. The method has practical application value for the intelligent classification of point clouds, and also has certain reference significance for the realization of assisted driving technology in unmanned driving. In this paper, the following is achieved:

(1) An improved convolutional neural network model is proposed for road traffic sign detection in panoramic images. The method is based on the YoloV3 deep learning model, adding a convolutional block attention module to the algorithm framework to enhance the saliency of the detection target in the image, improving the traditional K-means clustering algorithm by using the area intersection ratio between the target frame and the real frame as the distance loss function, generating a priori anchor frames instead of default values, and introducing Focal Loss as the loss function to solve the imbalance between the target and the background. In our study, the improved model is tested on TT100K dataset, and compared with YoloV3 before improvement, it is proved that the improved model in this study has higher accuracy.

(2) A method is proposed to accurately extract traffic signage from point clouds by combining image detection results. The method is divided into two steps. The first step is to locate the area where the traffic sign is located with the help of internal and external parameters of the image. The central projection method is used to extract the point cloud within a certain range near the target. The second step is to cluster the point clouds within the range using a region growing algorithm, and filter out the points that do not meet the requirements by intensity filtering and RANSAC plane fitting. Then, the dimensional characteristics of all the point clouds are analyzed based on PCA algorithm, and the traffic sign is finely extracted.

(3) Cross-comparison experiments were conducted from two aspects: whether the image is combined or not, and different traffic sign extraction methods. The experiments proved that the accuracy and recall rate of the traffic sign extraction method based on point cloud combined image proposed in this paper were significantly improved compared with other methods.

The overall structure of this paper is as follows: Section 1 explains the background of our research and summarizes the related work. Section 2 introduces the improved YOLOv3 algorithm. Section 3 describes the method of accurately extracting point cloud of traffic signs. Section 4 presents the analysis of experimental results. Finally, Section 5 presents the summary and future prospects.

## 2. Traffic Sign Detection

### 2.1. Improved Yolo Network

**Figure 1:**
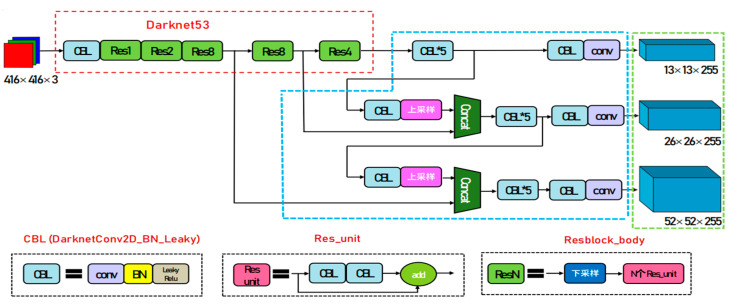
Schematic diagram of Yolov3 network structure.

Yolo network is a regression-based target detection algorithm that uses a feature extraction network to obtain the input image feature map, divides the input image into S×S grids, and if the center point of the target to be detected falls into a certain grid, the grid is responsible for detecting the target. The Yolov3 [34] network consists of the Backbone (red dashed area), Neck (blue dashed area), and Head (green dashed area). The input image is processed by Backbone to obtain feature maps of different scales, then processed by Neck to obtain deeper features, and finally sent to Head for detection. As shown in Figure 1, all the basic modules in the network consist of Convolutional Layer (Conv), Batch Normalization (BN), and LeakyRelu activation layers.

Since the Yolo series did not undergo further principal changes after version 3, this paper proposes an improved Yolo detection model based on YoloV3, combined with currently proven mainstream deep learning techniques. With specific improvements, including the introduction of a convolutional block attention module, the use of K-means to generate anchor boxes, and the use of GIoU and Focal Loss to improve the loss function.

#### 2.1.1. Convolutional Block Attention Module

Neural networks are designed to mimic the structure of human brain neurons, and the Convolutional Block Attention Module (CBAM) [35] is designed to mimic human attention. Adding an attention mechanism module is to add attention to input weight assignment, which can be divided into channel attention that focuses on pixel information and spatial attention that focuses on location information, etc. In this paper, we improve the performance of model detection by adding a CBAM, which fuses channel and spatial attention to the backbone network.

First, a channel attention mechanism module is generated to carry out the global maximum pooling and global average pooling of the input feature maps (H × W × C) to output two 1 × 1 × C feature maps. Then, they are fed into a two-layer mutually shared neural network MLP, and the output two feature maps are summarized and activated using the Sigmoid function to finally generate a 1 × 1 × C size channel attention module. The calculation formula is shown in Equation (1). where σ denotes the Sigmoid activation function, F represents the input feature maps, MLP denotes the shared network of two layers, the $W_{0}$ and $W_{1}$ denote the weight matrix in the MLP. $W_{0}$∈$R^{C/r\times C}$, $W_{1}$∈$R^{C\times C/r}$, where r is the decay rate, AvgPool, and MaxPool denote the average pooling and maximum pooling operations. $F_{avg}^{c}$ and $F_{max}^{c}$ operations denote the global average pooling and maximum average pooling operations of the channel attention mechanism.

(1) $$\begin{array}{c} M_{c}\left(F\right)=\sigma \left(MLP\left(AvgPool\left(F\right)\right)+MLP\left(MaxPool\left(F\right)\right)\right)\\ \;\;\;\;\;\;=\sigma \left(W_{1}\left(W_{0}\left(F_{avg}^{c}\right)\right)+W_{1}\left(W_{0}\left(F_{max}^{c}\right)\right)\right) \end{array}$$

After that, the original input feature map and the channel attention mechanism module are subjected to the homogeneous multiplication operation to obtain the feature map of the fused channel attention mechanism. The global average pooling and global maximum pooling operations are performed, respectively, to obtain two H × W × 1 feature maps, which are stitched together and then downscaled by the convolution operation. It is proved that the convolution kernel has the best effect with the size of 7 × 7 [35], which is activated by the Sigmoid function to generate a spatial attention mechanism module of size H × W × 1. The calculation formula is shown in Equation (2): $f^{7\times 7}$ represents the convolutional kernel of size 7 × 7 for the convolutional operation, $F_{avg}^{S}$ and $F_{max}^{S}$ operations represent the global average pooling and maximum average pooling operations of the spatial attention mechanism.

(2) $$\begin{array}{c} M_{S}\left(F\right)=\sigma \left(f^{7\times 7}\left(\left[AvgPool\left(F\right);MaxPool\left(F\right)\right]\right)\right)\\ \;\;=\sigma \left(f^{7\times 7}\left(\left[F_{avg}^{S};F_{max}^{S}\right]\right)\right) \end{array}$$

**Figure 2:**
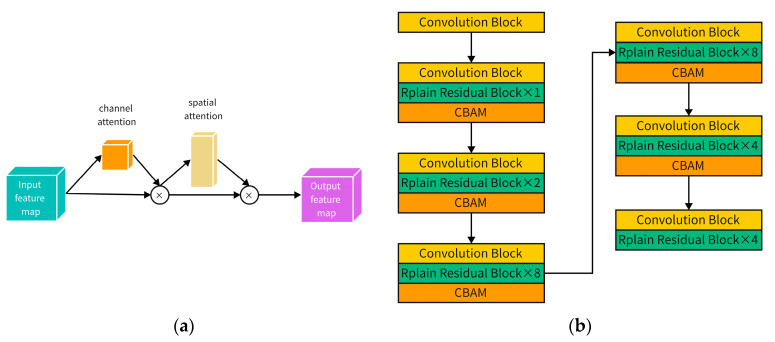
In this paper, a convolutional block attention mechanism module is added to the backbone region of the network, and a CBAM is added to each network residual module to be able to make full use of the effective information in the image and improve detection accuracy: (**a**) CBAM module structure; (**b**) Join the network residual module of CBAM.

Finally, the previously obtained fusion channel attention mechanism feature map is homogeneously multiplied with the spatial attention mechanism module to obtain the CBAM module, whose structure is shown in Figure 2.

#### 2.1.2. K-Means

The anchor box is used in Yolo as an a priori box to assist in predicting target boundaries. The anchor box is the shape and size of the most frequently occurring prediction box from all actual samples, and the default anchor box value is derived for the COCO dataset that can be used for large-scale target detection. A suitable anchor box can effectively improve localization accuracy and accelerate convergence. To make the anchor box size more representative of traffic signs, this paper proposes to use the K-means clustering algorithm to update the anchor value. If the Euclidean distance is used as the loss function, a larger target will generate a larger loss function compared to a smaller target, resulting in a larger error in the results of clustering. Therefore, this paper uses the Intersection Over Union (IoU) ratio between the prediction frame and the real frame instead of the Euclidean distance as the loss function distance measure; the larger the IoU, the smaller the loss, and the objective function of clustering is defined in Equation (3).

(3) $$F=min\overset{n}{\underset{i=1}{\sum }}\overset{k}{\underset{j=1}{\sum }}\left[1-\frac{truth_{i}\cap box_{j}}{truth_{i}\cup box_{j}}\right]$$

In the above equation, n is the number of samples; k is the number of selected clustering centers, which is generally set to 9; ${\mathrm{truth}}_{i}$ is the reference anchor box, ground truth; $box_{j}$ is the jth clustering center.

#### 2.1.3. Modified Loss Function

**Figure 3:**
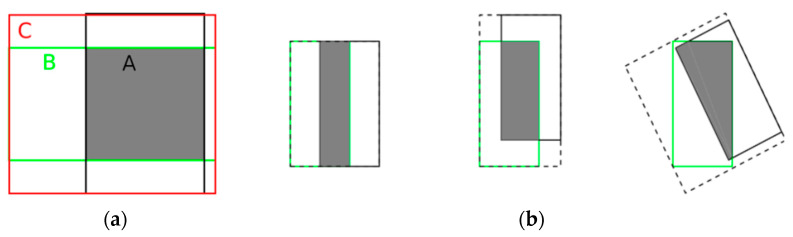
The parameters in the formula are shown in figure (**a**), A and B are two rectangular regions, C is the smallest external frame after the intersection of A and B, and C\(A ∪ B) indicates the area of C minus the area of A ∪ B. All three plots in (**b**) have the same IoU, but the GIoU decreases sequentially. It indicates that GIoU not only reflects the overlap between the prediction frame and the sign reference frame, but also considers their alignment, and the higher the alignment, the higher the GIoU value.

Bounding box regression is an important part of target detection, and metric loss calculated by IoU is used instead of regression loss in Yolo. There are some shortcomings in using IoU as a metric function: if there is no intersection between the reference anchor box and the prediction, the IoU value will be zero, which cannot reflect the distance between them at this time, and IoU cannot distinguish the alignment between two anchor boxes. So, this paper uses the generalized gradient intersection ratio (Generalized Intersection Over Union, GIoU) [36] to replace IoU as the loss function. As shown in Figure 3. The calculation formula is as in Equation (4).

(4) $$\mathrm{GIOU}=\frac{A\cap B}{A\cup B}-\frac{\left|C\backslash \left(A\cup B\right)\right|}{\left|C\right|}$$

In the process of loss function calculation of the network, the GIoU of the prediction frame and the standard reference frame are regarded as positive samples when it is greater than a certain threshold and negative samples when it is less than that threshold. However, the traffic sign data usually come from panoramic images or surveillance equipment, and the proportion occupied by the signage in the image is generally much smaller than the background proportion. For the problem of unbalanced positive and negative samples in the detection data, the loss function of the network is used instead of the original confidence loss by using Focal Loss, and the formula for calculating Focal Loss is shown in Equation (5).

(5) $$f\left(p,y\right)=\{\begin{array}{c} \;-\alpha {\left(1-p\right)}^{\gamma }log\left(p\right),\;\;\;\;\;\;\;\;\;\;\;y=1\\ \;-\left(1-\alpha \right)p^{\gamma }log\left(1-p\right),\;\;\;\;\;\;\;\;\;\;\;y=0 \end{array}$$

The final loss function consists of three components, as in the following equation.

(6) $$Loss=Loss_{position}-Loss_{confidence}+Loss_{class}$$

$Loss_{position},\;Loss_{confidence},Loss_{class}$ represents location loss, confidence loss, and classification loss. Among them, the confidence loss is modified by Focal Loss, and it is proved that the best detection effect is achieved when Focal Loss is introduced together with the adjustment of the GIoU threshold. The calculation formulae of localization loss, confidence loss, and classification loss are shown in Equations (7)–(9).

(7) $$\begin{array}{c} Loss_{position}=\left(2-w\times h\right)\sum_{i=0}^{S^{2}}\sum_{j=0}^{B}I_{ij}^{obj}\left[\left(t_{xi}-\hat{t_{xi}}\right)+\left(t_{yi}-\hat{t_{yi}}\right)\right]\\ \;\;\;\;+\left(2-w\times h\right)\sum_{i=0}^{S^{2}}\sum_{j=0}^{B}I_{ij}^{obj}\left[\left(t_{wi}-\hat{t_{wi}}\right)+\left(t_{hi}-\hat{t_{hi}}\right)\right] \end{array}$$

(8) $$\begin{array}{c} Loss_{confidence}=\sum_{i=0}^{S^{2}}\sum_{j=0}^{B}I_{ij}^{noobj}\left[\hat{C_{i}}\alpha (1-C_{i}{}^{\gamma }log\left(C_{i}\right)+\left(1-\hat{C_{i}}\right)\left(1-\alpha \right)C_{i}^{2}log\left(1-C_{i}\right)\right]\\ \;+\sum_{i=0}^{S^{2}}\sum_{j=0}^{B}I_{ij}^{obj}\left[\hat{C_{i}}\log\left(C_{i}\right)+\left(1-\hat{C_{i}}\right)\log\left(1-C_{i}\right)\right] \end{array}$$

(9) $$Loss_{class}=-\overset{S^{2}}{\underset{i=0}{\sum }}\overset{B}{\underset{j=0}{\sum }}I_{ij}^{obj}\underset{c\in n}{\sum }\left[\hat{p_{i}}\left(c\right)log(p_{i}\left(c\right)+\left(1-\hat{p_{i}}\left(c\right)\right)+\left(1-\alpha \right)log\left(1-p_{i}\left(c\right)\right)\right]$$

where w and h represents the width and height of the current grid; S denotes the number of grids and B denotes the number of prediction frames in which the current grid is located. $I_{ij}^{obj}$ and $I_{ij}^{noobj}$ denote the presence or absence of the target in the *i*th grid in the *j*th prediction frame, which takes the value of 0 or 1. $t_{xi},\;t_{yi},\;t_{wi},\;t_{hi}$ denotes the horizontal and vertical coordinates of the center point of the real target grid and the width and height of the grid.$\;\hat{t_{xi}},\hat{\;t_{yi}},\hat{\;t_{wi}},\;\hat{t_{hi}}$ denotes the horizontal and vertical coordinates of the center point of the predicted target grid and the width and height of the grid. $C_{i}$ and $C_{i}$ denote the actual and predicted confidence levels in the *i*th grid. $p_{i}\left(c\right)$ and $\hat{p_{i}}\left(c\right)$ denote the probability of the real box category and the probability of the predicted box in the *i*th grid. α is used to balance the samples and the γ denotes the rate of sample weight reduction.

### 2.2. Detection Result

#### 2.2.1. Experimental Data

The CSUST Chinese Traffic Sign Detection Benchmark (CCTSDB), produced by the Changsha University of Technology, is an extension of the CTSDB dataset. It is an expansion of the CTSDB dataset. At present, only part of the data is published, and there are three major categories of labeled data: indication signs, prohibition signs, and warning signs. The Tsinghua-Tencent traffic dataset (TT100K), jointly published by Tsinghua University and Tencent, features panoramas stitched together from six DSLR camera shots, capturing images from vehicles and shoulder-mounted devices at approximately 10-m intervals. TT100K selects 10 areas from five different cities in China (including each city’s urban). The TT100K dataset provides a more detailed classification of traffic signs, but the dataset also suffers from an unbalanced number of labeled samples.

In this paper, we propose using the TT100K dataset as the main dataset for training. Forty-six categories are labeled and classified in the original dataset, and the precise identification of each category is not the focus of this paper, so the labeled categories are divided into three categories: ban signs, instruction signs, and warning signs. To balance the problem of a serious imbalance in the number of samples, the CCTSDB dataset is introduced to expand the samples, and the distribution of the balanced dataset is shown in Table 1.

#### 2.2.2. Refine Results

**Figure 4:**
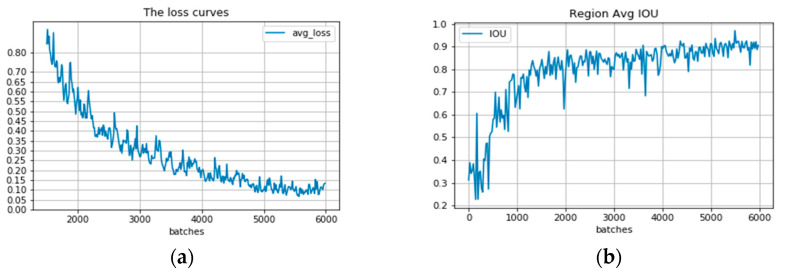
The model is trained for 6000 iterations, and in figure (**a**), it can be seen that the trend of the model’s Loss is oscillating steadily down, and the final Loss value is stable at about 0.1; in figure (**b**), it can be found that the final average cross-merge ratio of the model has also converged. In summary, after 6000 iterations, all the indexes have reached a stable state.

**Figure 5:**
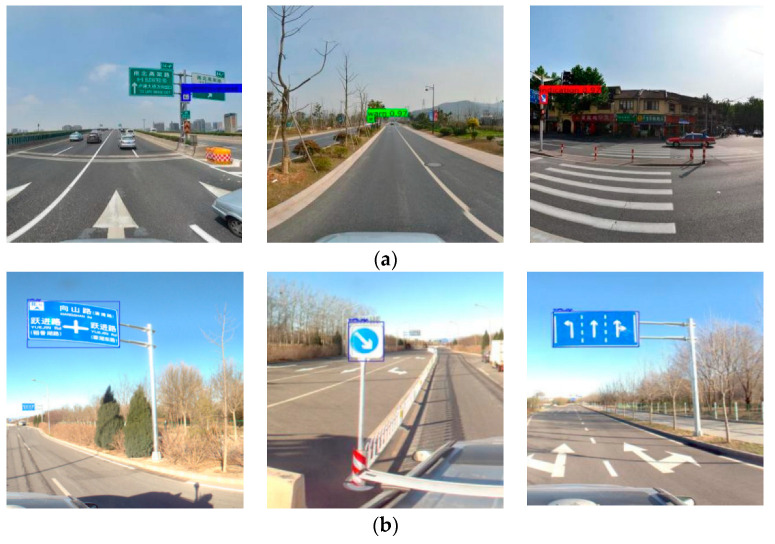
(**a**) Test set detection results; (**b**) Panoramic detection effect.

The model can reach a stable convergence state in the performance of the convolutional neural network model when it is trained. In this paper, the training log of the model is recorded and visualized in the output, and the output metrics include Loss value and average IoU, as shown in Figure 4. The improved model is tested on the test set and panoramic image, respectively, and the results are shown in Figure 5.

In this paper, precision and recall are chosen as evaluation metrics, the former reflecting the accuracy of classification, and the latter reflecting the degree of completeness of detection. *TP* indicates the number of correctly detected targets, *FP* indicates the number of incorrectly detected targets, and *FN* indicates the number of missed detections. The correct rate Precision and recall rate Recall are defined as shown in Equations (10) and (11).

(10) $$\left|Precision=\frac{TP}{TP+FP}\right|$$

(11) $$\left|Recall=\frac{TP}{TP+FN}\right|$$

As shown in Table 2, the model recall of the method used in this paper is 1.8% higher than the original Yolov3, and the overall accuracy value is 1.4% higher. Specifically, the overall detection accuracy of ban signs and instruction signs will be higher, reaching 86.3% and 83.7%, with 1.6% and 1.8% accuracy improvement compared to the original method, while the accuracy of warning signs only improves by 0.9% compared to the original method, reaching 80.2%. This may due to the complex pattern of warning signs, which is more difficult to distinguish.

## 3. Traffic Sign Positioning Extraction

### 3.1. Image Coordinate Transfer to Point Cloud

**Figure 6:**
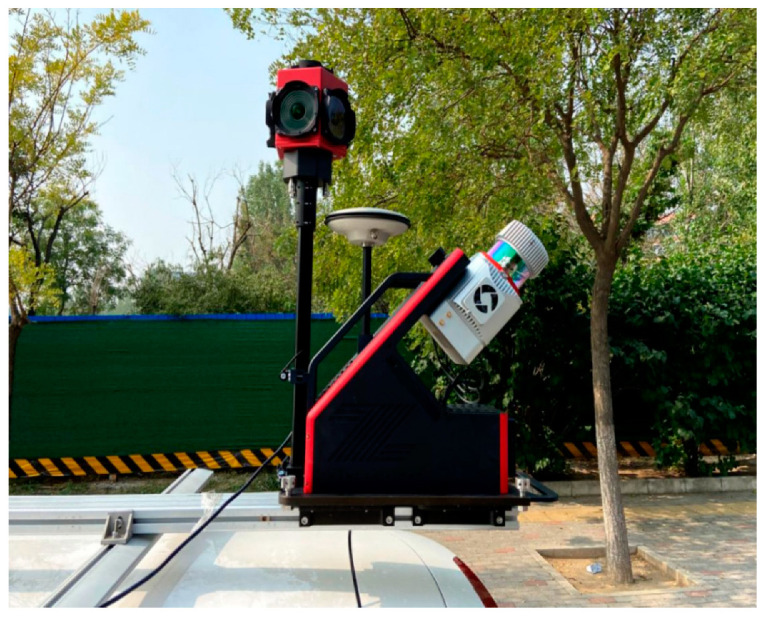
The vehicle-mounted laser measurement system SZT-R1000.

The high precision conversion of pixel coordinates to point cloud coordinates is a prerequisite for making full use of image detection results. Next, we will introduce the transformation process from pixel coordinates to point cloud coordinates in detail, and carry out crude extraction of traffic signs based on this. The vehicle-mounted laser measurement system(Figure 6) is equipped with a panoramic camera that continuously captures images during travel. Meanwhile, in the LIDAR radar system, the laser range scanning unit continuously emits microwaves to obtain distance information, the Differential Global Position System (DGPS) obtains position information, and the Inertial Measurement Unit (IMU) obtains attitude information. The geometric relationship between the camera coordinate system and the corresponding ground points in the geodetic coordinate system can be determined from the internal and external orientation elements of the image. The image acquired by the camera can be matched with the point cloud to give color information.

#### 3.1.1. Obtaining Depth Map

The external attitude parameters of each frame of panoramic image can be obtained by the combination navigation of panoramic camera and lidar system. First, the world coordinate system is converted to the camera coordinate system, which is based on the following principle:

(12) $$\left[\begin{array}{c} X_{\mathrm{cam}}\\ Y_{\mathrm{cam}}\\ Z_{\mathrm{cam}} \end{array}\right]=\;R\left[\begin{array}{c} X_{\mathrm{word}}\\ Y_{\mathrm{word}}\\ Z_{\mathrm{word}} \end{array}\right]+\left[\begin{array}{c} T_{x}\\ T_{y}\\ T_{z} \end{array}\right]$$

(13) $$R=\left[\begin{array}{ccc} 1 & 0 & 0\\ 0 & \cos\beta & \sin\beta \\ 0 & -\sin\beta & \cos\beta \end{array}\right]\left[\begin{array}{ccc} 0 & \cos\gamma & -\sin\gamma \\ 0 & 1 & 0\\ 0 & \sin\gamma & \cos\gamma \end{array}\right]\left[\begin{array}{ccc} \cos\alpha & \sin\alpha & 0\\ -\sin\alpha & \cos\alpha & 0\\ 0 & 0 & 1 \end{array}\right]$$

In the above equation, the ${\;X}_{\mathrm{cam}}$, ${\;Y}_{\mathrm{cam}}$, $Z_{\mathrm{cam}}$ represent the coordinates under the camera coordinate system. u, v are the pixel coordinates of the point. $X_{\mathrm{word}}$, $Y_{\mathrm{word}}$ and ${\;Z}_{\mathrm{word}}$ denote the world coordinates of the point cloud.$T_{x}$, $T_{y}$ and ${\;T}_{z}$ denote the offset of the camera with respect to the origin of the world coordinate system. α, β and γ denote the three attitude angles of the outer azimuth element parameters.

The depth image requires the acquisition of distance information for each pixel point, which is calculated by converting to the pixel coordinate system with the help of the spherical coordinate system.

(14) $$u\;=\frac{\arccos\left(\frac{Z_{\mathrm{cam}}}{r}\right)}{\pi }H$$

(15) $$v\;=\left[\left(\frac{\;\mathrm{atan}2\left(Y_{\mathrm{cam}},X_{\mathrm{cam}}\right)}{2\pi }+0.5\right)W\right]$$

In the above equation, the u, v denotes the pixel coordinates of the point; and r denotes the distance from the center of photography to the target point; calculated from the camera position and the position parameters in the external camera reference. W and H are the width and height of the panoramic image.

Based on the above theory, the coordinates of the image point of the point cloud in the panoramic image and the distance from the point cloud to the center of photography are calculated, from which the pixel value of the depth image is calculated to generate the final depth image, and the pixel value is calculated by the following formula.

(16) $$D_{i}=\frac{r_{i}\;}{d\times 255}$$

**Figure 7:**
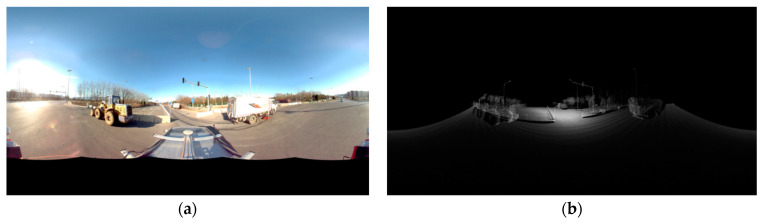
Figure (**a**) is a panoramic photo of the Fourth Ring Road in Beijing. By adding the distance constraint, meaningless point cloud information can be excluded and the computational effort can be reduced, the generated depth image is shown in figure (**b**).

In the above equation, $D_{i}$ denotes the pixel value of each pixel point in the depth image, $r_{i}$ denotes the Euclidean distance from the point cloud to the photography center, d denotes the distance constraint for generating the depth image, and the pixel values larger than this value from the center of photography will be ignored. The generated depth map is shown in Figure 7.

#### 3.1.2. Image Projection to Point Cloud

The conversion from pixel coordinates to LIDAR coordinates can be carried out in two steps: Firstly, converting the pixel coordinate system to the polar coordinate system, and then converting the polar coordinate system to the LIDAR coordinate system, as calculated by the following equation.

(17) $$\left[\begin{array}{c} X_{p}\\ Y_{p}\\ Z_{p} \end{array}\right]=\left[\begin{array}{c} \rho \sin\theta \sin\varphi \\ \rho \sin\theta \cos\varphi \\ \rho \cos\theta \end{array}\right]$$

(18) $$\left[\begin{array}{c} \theta \\ \varphi \\ \rho \end{array}\right]=\left[\begin{array}{c} \pi \;\times \frac{u}{H}\\ \;\pi \;\times \frac{v-H}{H}\\ k\;\left(1-\;D/d\right) \end{array}\right]$$

Equation (17) represents the conversion from the polar coordinate system to the LIDAR coordinate system. $X_{p}$, $Y_{p}$ and ${\;Z}_{p}$ denote the three-dimensional coordinates in the laser coordinate system. ρ, θ and φ denote the sphere in the three-dimensional coordinates value. Equation (18) represents the conversion from the pixel coordinate system to the polar coordinate system. u and v denote the pixel coordinates, k is the conversion constant, and D denotes the depth image pixel value.

The main process of the conversion of LiDAR coordinates to world coordinates is as follows. Firstly, the points of the LiDAR coordinate system are transferred to the carrier coordinate system. Then, the conversion matrix from carrier coordinates to geodetic coordinates is generated according to the external orientation parameter file. Finally, the position of the carrier center under the geodetic coordinate system is added. The calculation process is as follows:

(19) $$\left[\begin{array}{c} X_{G}\\ Y_{G}\\ Z_{G} \end{array}\right]=\left[\begin{array}{c} X_{B}\\ Y_{B}\\ Z_{B} \end{array}\right]+R\left[\left[\begin{array}{c} X_{p}\\ Y_{p}\\ Z_{p} \end{array}\right]+\left[\begin{array}{c} x_{0}\\ y_{0}\\ z_{0} \end{array}\right]\;\right]$$

$X_{G}$, $Y_{G}$ and $Z_{G}$ denote the three-dimensional coordinates in geodesic coordinates. ${\;X}_{p}$, $Y_{p}$ and $Z_{p}$ represent the three-dimensional coordinates under the LiDAR coordinate system. ${\;X}_{B}$, $Y_{B}$ and $Z_{B}$ are the offset of the carrier center in the geodetic coordinate system. $x_{0}$, $y_{0}$ and $z_{0}$ are the offset of the lidar coordinate origin in the carrier coordinate system. R is the conversion matrix from the carrier coordinate system to the geodesic coordinate system, which is defined as shown in Equation (20).

(20) $$R\;=R_{\varphi }\;R_{\omega }\;R_{\kappa }$$

**Figure 8:**
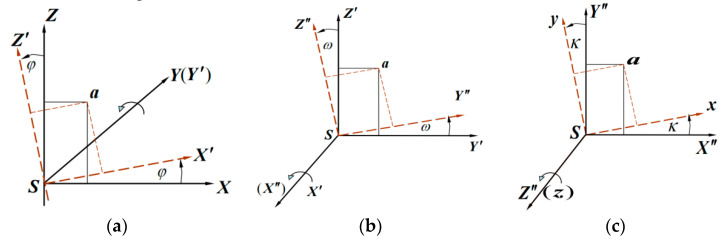
(**a**) To perform the conversion, first wind the original image spatial coordinates XYZ−S under the Y axis φ to obtain the coordinate system $\;X^{\prime }Y^{\prime }Z^{\prime }-S$; (**b**) Then rotate around $\;X^{\prime }$ axis to obtain the coordinate system $\;X^{\prime }Y^{\prime }Z^{\prime }-S$; (**c**) Finally rotate around $\;Z^{\prime \prime }$. The final spatial coordinate system is obtained by rotating around the axis, as shown in the figure below.

The conversion matrix consists of three angular elements φ, ω, κ in the outer orientation element. The pitch angle φ (pitch) indicates the angle between the carrier coordinate system axis and the ground plane; the carrier lift is positive. (As shown in Panel a of Figure 8). The side roll angle ω (roll) indicates the angle between the carrier symmetry plane and the carrier vertical axis plumb plane; the right is positive. (As shown in Panel b of Figure 8). The yaw angle κ (yaw) indicates the angle between the projection of the carrier axis on the horizontal plane and the ground axis; the right is positive. (As shown in Panel c of Figure 8).

The rotation matrices $R_{\varphi }$, $R_{\omega }$ and $R_{\kappa }$ can be expressed, respectively, as follows:

(21) $$R_{\varphi }=\left[\begin{array}{ccc} \cos\varphi & 0 & -\sin\varphi \\ 0 & 1 & 0\\ \sin\varphi & 0 & \cos\varphi \end{array}\right]$$

(22) $$R_{\omega }=\left[\begin{array}{ccc} 1 & 0 & 0\\ 0 & \cos\omega & -\sin\omega \\ 0 & \sin\omega & \cos\omega \end{array}\right]$$

(23) $$R_{\kappa }=\left[\begin{array}{ccc} \cos\kappa & 0 & -\sin\varphi \\ 0 & 1 & 0\\ \sin\varphi & 0 & \cos\varphi \end{array}\right]$$

From Equation (20)–(23), the conversion matrix expression is obtained as follows:

(24) $$R\;=\left[\begin{array}{ccc} \cos\varphi \cos\kappa -\sin\varphi \sin\omega \sin\kappa & -\sin\varphi \cos\omega & \sin\varphi \cos\kappa +\cos\varphi \sin\omega \sin\kappa \\ -\cos\varphi \sin\kappa -\sin\varphi \sin\omega \cos\kappa & \cos\omega \cos\kappa & -\sin\varphi \sin\kappa +\cos\varphi \sin\omega \cos\kappa \\ -\sin\varphi \cos\omega & -\sin\omega & \cos\varphi \cos\omega \end{array}\right]$$

### 3.2. Region of Interest Extraction

**Figure 9:**
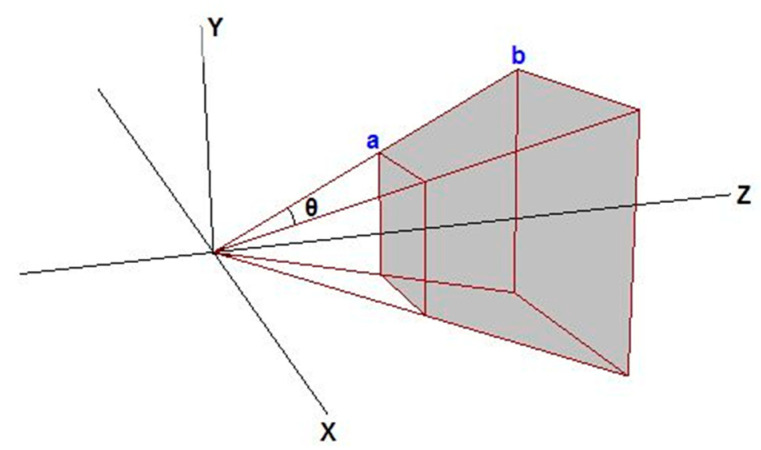
As shown in Figure 9, the photography center is placed at the origin of the coordinate system, and a projection beam is launched to the four 3D coordinates of the prediction box to form a conical projection area with a rectangular bottom. In this process, the scope of the cone region will be set according to the actual situation, and it will not be allowed to extend indefinitely. The shaded area is the region of interest. Using this method to extract the region of interest can fully contain the detection target, ensuring that no omissions occur.

After obtaining the detection frame geodesic coordinates, there are still challenges in how to make full use of the 2D detection results to extract the point cloud data. Firstly, the prediction box in the image is in the two-dimensional plane, which can provide four effective coordinates, and at least eight vertex coordinates are needed to establish the bounding box in 3D. Secondly, the vertex coordinates of the prediction frame may be located on the target or outside the target; although the feature point is on the photographic beam consisting of the photographic center and pixel coordinates, it is impossible to determine the exact range. A common practice is to determine the range of point clouds by means of central projection, which means that the light source is regarded as a point and the light is scattered outward to the projection surface. The central projection is shown in Figure 9.

**Figure 10:**
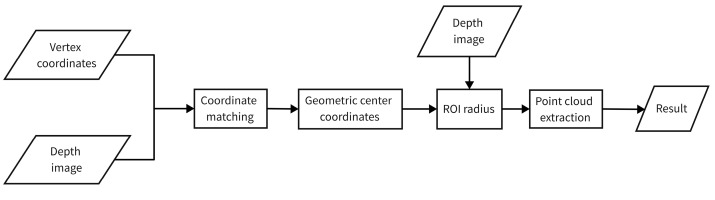
Flow of region of interest extraction with image detection results.

In this paper, based on the image detection results, we use image depth information for point cloud signage area of interest extraction. After aligning the prediction frame vertex coordinates with the depth image, the approximate geometric center of the detected traffic signage is located. Then, this center is taken as the origin, the depth image pixel value is combined to determine the signage range, and the extraction radius is determined according to this range. With no restriction in the vertical ground direction, the ground direction takes the radius as the threshold for region of interest extraction. The main process is shown in Figure 10.

**Figure 11:**
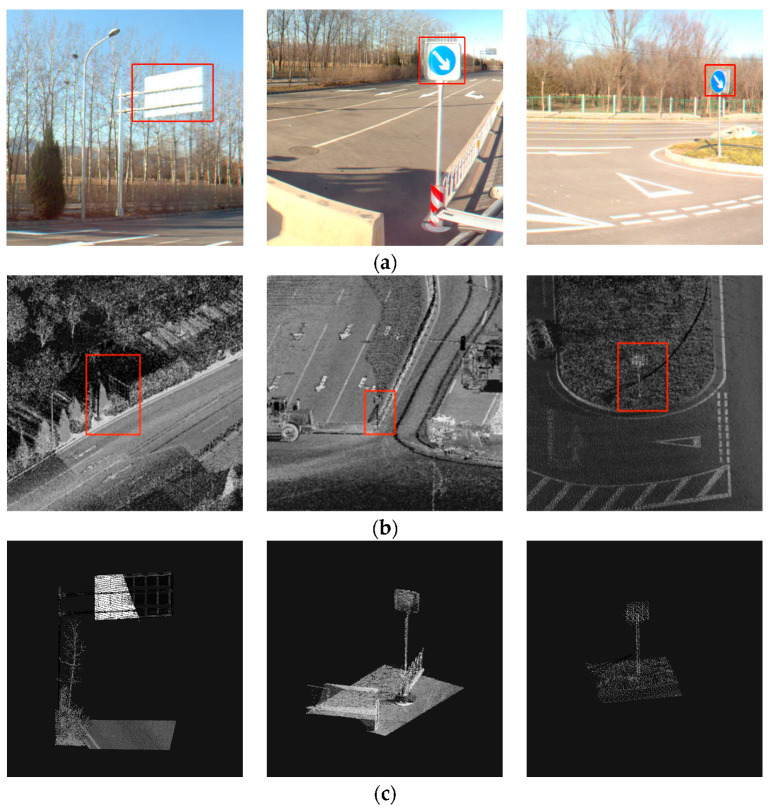
Point cloud area after positioning and extraction. (**a**) Panoramic image detection results; (**b**) Raw data; (**c**) post-positioning area.

The target area extracted according to the coordinate conversion result is shown in Figure 11. The extraction threshold is determined by extending the approximate center point of the traffic signage outward, which not only ensures the integrity of the traffic signage itself but also ensures that there is only one target object in the extraction area. The scope of the extracted data is greatly reduced to facilitate the subsequent refined extraction of traffic signs.

### 3.3. Refined Extraction

Traffic signage has the following characteristics in the point cloud data: (1) traffic general signage is suspended from the pole, above the ground, and perpendicular to the ground. (2) Traffic signage belongs to the façade, with a regular geometric shape, generally rectangular or triangular. (3) In order to ensure the safety of traffic, the state requires the surface of traffic signs to be sprayed with high-reflectivity materials, and the national safety production standard has a minimum value of reflectivity for traffic sign equipment, including poles.

**Figure 12:**
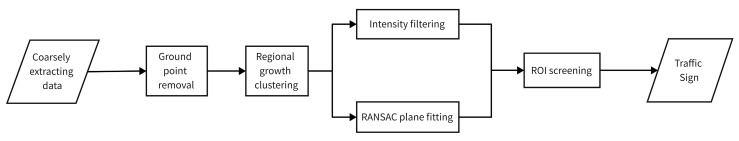
Signage extraction technical route.

Combining the above characteristics, this paper adopts the point cloud traffic sign extraction by combining intensity features and morphological features. Although the atmospheric attenuation and laser acquisition incidence angle will affect the reflection intensity of the point cloud data, this feature can still be used to distinguish the traffic sign from most of the features. At the same time, the RANSAC method is used to fit the plane where the traffic sign is located and identify the traffic sign by the dimensional information. The technical route is shown in Figure 12.

#### 3.3.1. Ground Points Removal

**Figure 13:**
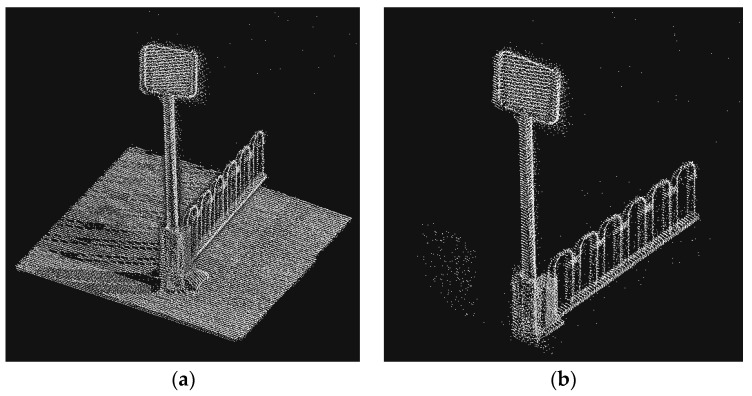
The contrast of point clouds before (**a**) and after (**b**) the ground is removed.

In this paper, we use the Cloth Simulation Filter (CSF) proposed by Wuming Zhang et al. [37]. The region of interest extracted in this paper has the characteristics of high accuracy and a small range; the overall area is relatively flat, so it meets the conditions of the application of this algorithm. The basic idea of the CSF algorithm is to cover a large piece of fabric on top of the inverted point cloud after removing isolated points. After fully considering the influence of external driving factors (gravity, collision) and internal driving factors (interaction between particles inside the fabric), the final fallen fabric can represent the current terrain. As shown in Figure 13, the distance relationship between the fabric and the inverted point cloud is used to determine whether it is a ground point or not. The algorithm has a strong generalization ability, few parameter settings, and has a good extraction effect under a non-steep slope area.

#### 3.3.2. Regional Growth Clustering

Due to the small amount of data in the target area, it is not suitable to use the RANSAC algorithm directly. Traffic signage has the characteristics of regular shape and obvious contour, and has obvious distance from other features. This paper adopts the region growing algorithm to perform point cloud clustering; the segmented region is used to establish ROI with the principle of closest neighbor.

The region growing algorithm is originally used in the field of image cutting, which considers that the object has a similar property in the same region and establishes the initial region by this property. From the initial region, the data with the same property are gradually grouped into the region, so that the segmentation of the region can be realized [38]. Since the point cloud data is in three-dimensional space, it has direction itself, so the direction information can be regarded as the target property. Information on the direction is measured by the smoothness between two points, which is expressed by the normal angle. When the normal angle between the points in the initial region and the neighboring points meets the specified requirements, both points belong to part of the smooth surface. The algorithm proceeds by iteration, and the output data structure is an array after clustering, thus completing the segmentation of the 3D point cloud.

**Figure 14:**
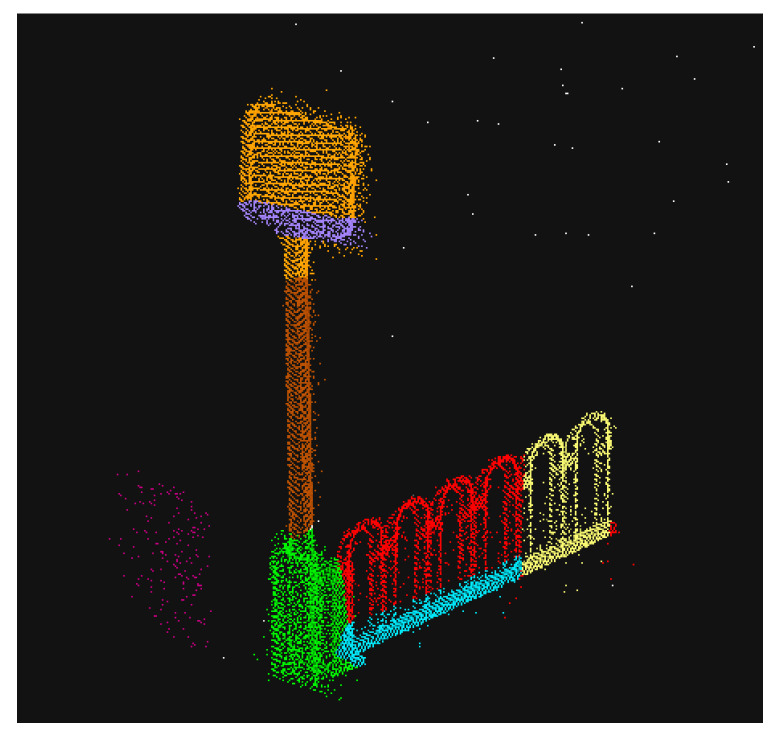
Point cloud growth clustering segmentation results.

The main process of this algorithm is as follows:

(1) All data points are sorted according to the size of the curvature; the point with the smallest curvature value is selected as the initial seed point, and the selected point is added to the seed set and the current region is marked.

(2) The normal direction of the seed point is calculated and the normal direction of each neighbor is determined in turn through the search. If the angle between the normal direction of the neighbor and the normal direction of the seed point is less than the threshold, the nearest neighbor is added to the current region.

(3) The curvature value of each nearest neighbor of the current seed point is calculated. If the curvature value is less than the given threshold, the nearest neighbor point is added to the seed set and the current seed point is removed from the seed array. The above steps are repeated until the seed array is empty.

(4) Steps (2) and (3) are repeated until all areas are marked and the final point cloud segmentation result is obtained as shown in Figure 14.

The advantages of the region growing algorithm are that it is more flexible to use, it can set the growing rules according to the actual scene, it has a better effect on segmenting the area around the seed point, and it is more efficient. However, the algorithm also has some limitations: when the connectivity of the region to be segmented is poor, the data are easy to segment into a small closed region, and the algorithm is susceptible to noise infection and has high time complexity. In addition to curvature, the region growth algorithm can also be based on color segmentation, which is the same principle as the method based on curvature. The basic idea is to divide the points set with similar colors in the same region into the same class.

#### 3.3.3. Intensity Filtering and RANSAC Planar Fitting

The point cloud data measured by the laser scanner can not only obtain the coordinate information and color information, but also obtain the reflection intensity information of each point. The reflection intensity information is not only related to the reflection ability of the target but is also affected by the scanning distance, angle, atmospheric conditions, and other factors. For the same point cloud data, the intensity of the laser echo is closely related to the material of the object being measured, and the higher the reflectivity of the object itself, the higher the reflectivity value of the point cloud. In order to ensure safety, traffic signs are sprayed with paint that increases the reflectivity, so they have high reflectivity in the point cloud data, and some non-target areas can be removed accordingly. Since the traffic signs themselves are outdoors for a long time, the paint will inevitably be damaged and fall off, which may lead to the reflectivity of the points on the traffic signs not reaching the standard level. So, our paper uses the clusters obtained from the area growth as the unit for filtering. The specific approach is to set up a reflection intensity threshold and to count the point clouds in each cluster. If the majority of the point cloud intensities are lower than this threshold, it is considered that the cluster is not a cluster containing traffic signage. The threshold is determined without the purpose of separating traffic signage, but a large number of experiments are needed to determine the best threshold. Most of the ground appendages can be filtered after point cloud filtering, such as stone piers and fences, etc. What remains are mainly traffic sign poles, their appurtenant pole objects, and some highway barriers near the traffic, which can be fitted with planar features for further screening.

The nature of estimating object parameters from sampled points has a long history of research, where the RANSAC algorithm [39] has high robustness to noise in point cloud data; therefore, it is often used to extract local models from mixed data. The random sampling consistency algorithm (Random Sample Consensus (RANSAC) is an effective and robust estimation algorithm. The RANSAC algorithm considers that the data are composed of valid data with small deviations. These points are called intra-local points, and the distribution of intra-local points can be explained using model parameters. However, data points with large deviations that cannot be applied to the model are classified as extra-local points, such as noisy extremes, incorrect measurements, etc., as well as noise points. The basic idea of this method is to establish a mathematical model to obtain the model distribution parameters of local points through a set of sample data sets containing various external points and noise points. Specifically, a hypothetical model is established, and if the initial data meet the model judgment conditions, it is expanded using a consistent data set. If there are enough point distributions that can be explained using the model parameters, the hypothetical model is reasonable enough, which is actually an idea of using the model to fit the data.

**Figure 15:**
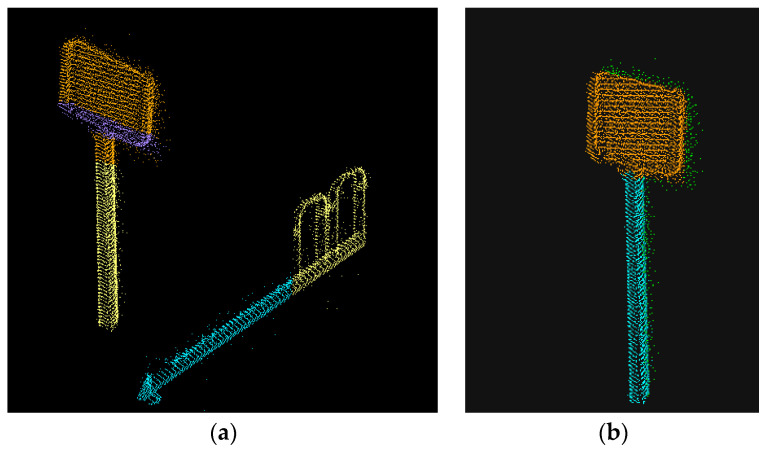
(**a**) After intensity filtering, most ground appendages can be filtered, traffic signs and their attached poles, and some highway barriers are retained. (**b**)After further screening by RANSAC fitting plane features, only traffic signs and their attached rods remain.

Traffic signage has an obvious faceted geometric shape in the point cloud data. In this paper, we use the RANSAC algorithm to fit the plane to the point cloud data in the extracted area and compare the enclosing box where the non-facets are located with the clustered results. As shown in Figure 15. When most points in a coarse class cluster are in the enclosing box of non-facets, it is considered that the coarse class cluster does not belong to the traffic signage. Combining the parameters of the laser scanner and the characteristics of the traffic signage, the number of data points constituting the traffic signage should be greater than one hundred, and those that do not meet the conditions should also be eliminated.

### 3.4. Dimensional Feature

#### 3.4.1. Calculating Eigenvalues

After processing in the previous sections, traffic signs and other features can be distinguished in most scenes. To further increase the accuracy, our paper uses the principal component analysis to calculate the dimensional features.

Principal Components Analysis (PCA) is a common method of dimensionality reduction, which aims to determine the variables that are not linearly related to each other by the correlation between data with the help of mathematical operations. Additionally, it is arranged in order of decreasing variance. While keeping the total variance constant, each group of variables is called the first principal component, the second principal component, and so on. The variables between each principal component are not correlated.

The covariance reflects the mutual relationship between two sets of variables; greater than zero means they are positively correlated, less than zero means they are negatively correlated, and equal to zero means they are independent from each other. After obtaining the covariance matrix formula for each point, the covariance matrix of its neighboring points is calculated as follows:

(25) $$M\;=\frac{1}{n}\overset{n}{\underset{i=1}{\sum }}\left(c_{i}-\bar{c}\right){\left(c_{i}-\bar{c}\right)}^{T}$$

where M is a 3 × 3 covariance matrix, and $c_{i}$ denotes the points in the neighborhood, $\bar{c}$ denotes the average coordinates in the neighborhood, n denotes the number of points, and further the eigen decomposition of M.

(26) $$M\;=\left[e_{1}{\;e}_{2\;}{\;e}_{3}\right]\left[\begin{array}{ccc} \lambda_{1} & 0 & 0\\ 0 & \lambda_{2} & 0\\ 0 & 0 & \lambda_{3} \end{array}\right]\left[\begin{array}{c} e_{1}{}^{T}\\ e_{2}{}^{T}\\ e_{3}{}^{T} \end{array}\right]$$

where $\lambda_{1}$, $\lambda_{2}$ and $\lambda_{3}$ denote the eigenvalues sorted from largest to smallest and $e_{1}$, $e_{2}$ and $e_{3}$ are the eigen vectors corresponding to the three eigenvalues.

#### 3.4.2. Dimensional Analysis

After calculating the eigenvalues, the dimensional features can be defined.

(27) $$a_{1D}=\frac{\sqrt{\lambda_{1}-\lambda_{2}}}{\sqrt{\lambda_{1}}},a_{2D}=\frac{\sqrt{\lambda_{2}-\lambda_{3}}}{\sqrt{\lambda_{1}}},a_{3D}=\frac{\sqrt{\lambda_{3}}}{\sqrt{\lambda_{1}}}$$

From Equation (28), it can be seen that when the first principal component is much larger than the second and third principal components, the $a_{1D}$ maximum. When the first principal component is close to the second principal component and much larger than the third principal component, $a_{2D}$ is the maximum. When the first two and three principal components are close to each other, the first two or three principal components are close to each other and the maximum is defined. Accordingly, the dimensional characteristics of the data points can be defined as follows:

(28) $$D=argMax\left(a_{iD}\right)$$

where *i* ∈ {1, 2, 3}, the above equation means that the dimension D of the point is the value of i when $a_{iD}$ is the maximum value. Combined with the analysis of Equation (28), the scanned points can be classified into three categories: linear points (D = 1), planar points (D = 2), and discrete points (D = 3).

**Figure 16:**
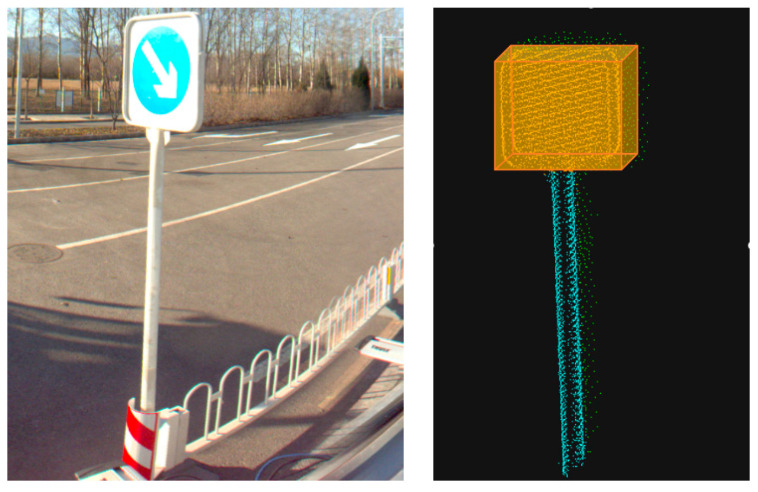
After the dimension characteristic judgment, the class cluster whose dimensional features are closest to plane features is screened out.

Since the RANSAC algorithm is more obviously influenced by parameters, it may fit objects with less obvious face features to a plane. As shown in Figure 13, which can be avoided by adding the judgment of dimensional features. The point cloud data after growth clustering is processed by intensity filtering and RANSAC screening; if there is still more than one cluster to be selected, the points in all clustering clusters are considered to be dimensional. Suppose there are two clusters A and B. If the dimensional mean $\bar{D_{a}}$ of all points in cluster A is closer to D = 2 than the $\bar{D_{b}}$, since there is only one traffic signage in the area of interest, A can be judged as traffic signage. Meanwhile, in order to further refine the contour of the traffic signage, the anomalous points of dimensional features in the clustering cluster are removed. The results are shown in Figure 16.

## 4. Discussion

### 4.1. Accuracy Analysis

In this paper, the reflection intensity-based extraction method and RANSAC-based extraction method are used for comparison. The reflection intensity-based extraction method extracts traffic signage based on the manually set intensity range; the RANSAC-based extraction method goes to extract the faceted area as traffic signage based on the manually set area region. The comparison experiments in this paper are divided into two aspects: whether to combine images and different traffic signage extraction methods. Therefore, there are five groups of experiments: (1) extraction based on reflection intensity for the whole point cloud; (2) extraction based on RANSAC for the whole point cloud; (3) extraction based on reflection intensity method combined with image positioning; (4) extraction based on RANSAC method combined with image positioning; (5) using the method of this paper.

In Table 3, Method I and Method III, Method II, and Method IV can form two sets of control tests, illustrating that locating traffic signs by image detection before performing traffic sign extraction can bring a huge improvement to the accuracy and recall rate. This is due to the elimination of most other easily confusing objects after establishing a reasonable target area. Method III and Method V, and Method IV and Method V can form two more sets of control tests, which illustrate that the traffic sign extraction method proposed in this paper has a better extraction effect compared with the reflection intensity-based method or the RANSAC method.

### 4.2. Visual Analysis

In this section, the original training set is reprogrammed using an improved Yolo network, and the panoramic images collected by the laser system on the South Fourth Ring Road of Beijing are used as an expanded dataset to include more indication signs on top of the original dataset. The overall process of the proposed method to extract traffic signage in the target point cloud range goes through several steps, including region growth clustering segmentation, ground point removal, intensity filtering, and dimensional feature screening.

**Figure 17:**
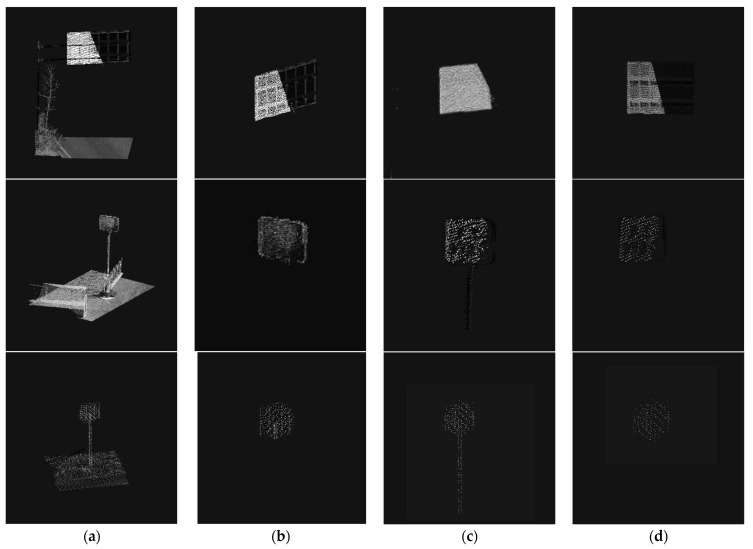
Comparison of our method with other methods: (**a**) Pre-Extraction Data; (**b**) Our Methodology; (**c**) Combined Imaging + RANSAC; (**d**) Combined image + reflection intensity. RANCAR algorithm incorrectly fits the traffic sign bar plane and extracts it. As traffic signs are exposed all year round, their reflective materials fall off, and the point clouds obtained by extracting traffic signs with intensity information are sparse or even missing.

The effect of traffic sign extraction in this paper is shown in Figure 17. It can be seen that the method in this paper achieves good extraction results for traffic signs in various scenes. It should be noted that although it may be difficult to detect in some images due to factors such as lighting, angle, and occlusion, the panoramic image is obtained by continuous shooting during the driving process, and the same target is shot at multiple angles, so it generally does not affect the recognition of this target.

## 5. Conclusions and Future Research Directions

Traffic signs play an important regulatory role in the process of road driving, and the correct identification of traffic signs is an important guarantee for driving safety. Extracting traffic signs accurately in complex and changing road scenes is a difficult problem. In this paper, we analyze the existing traffic sign extraction methods and propose a traffic sign extraction method that combines image detection and point cloud segmentation. In order to improve the signage detection accuracy in images, three aspects of the YoloV3 detection model are improved: network structure, prediction frame size, and loss function. The model is validated using domestic public road traffic datasets, and the experiments prove that the model has higher accuracy compared with the original model.

A localization method of traffic signage in LiDAR data is proposed for the panoramic images, and point cloud data acquired by vehicle-mounted mobile laser scanning equipment. The method can make full use of the detection results of deep learning in road images to accurately locate the range of traffic signage in the point cloud data. Then, it further combines the depth image to determine the extraction range and obtain the point cloud region where the traffic sign is located. After, the acquired point cloud region is subjected to a CSF filtering algorithm to remove the ground. The clusters are clustered using the region growing method, and the clustered target clusters are filtered using intensity filtering and RANSAC plane fitting to narrow the target range. For the remaining coarse class clusters, traffic signage is extracted using dimensional features based on the PCA method, while noise points with abnormal dimensionality are removed. In comparison with other commonly used point cloud segmentation extraction methods, it has a better extraction effect.

However, there are still some problems that need to be improved or solved. The method in this paper is harsh on data acquisition, which needs to rely on the external parameters of each panoramic photo derived from the conversion of the inertial guidance system. These parameter files are used as the initial data to realize the whole process, which has high requirements on data acquisition, cannot be realized solely by point cloud data, and the universality needs to be enhanced. Our method relies on visual detection results to extract point cloud, and the final accuracy is affected by the deep learning algorithm. Manually marking traffic signs in a 3D scene takes time and effort. The point cloud data extracted in this paper can provide a dataset for extracting traffic signs directly from 3D data.

## Figures and Tables

**Table 1:** Sample balance comparison.

Category	Before Balance	After the Balance
Prohibition signs	18,317	18,317
Indicator signs	4989	7631
Warning Signs	1396	5317

**Table 2:** Comparison of model results.

	Accuracy (%)			Overall Accuracy (%)	Recall Rate (%)
	Prohibited Signs	Indicator Signs	Warning Signs		
Yolov3	84.7	81.9	79.3	82.0	86.3
Ours	86.3	83.7	80.2	83.4	88.1

**Table 3:** Accuracy analysis results.

Method	Accuracy (%)	Recall Rate (%)
Extraction method based on reflection intensity	7.5	28.6
RANSAC-based extraction method	2.8	12.9
Combined Image + Reflection Intensity	76.3	94.7
Combined Imaging + RANSAC	87.6	94.7
Methodology of this article	97.8	94.7

## Data Availability

Not applicable.
